# Association of 5-hydroxytryptamine 3 receptor antagonists with prognosis in patients with advanced hepatocellular carcinoma: A propensity score-matched study

**DOI:** 10.1371/journal.pone.0359798

**Published:** 2026-10-08

**Authors:** Xiaoge Zhang, Xiaoyun Wang, Na Li, Qunying Han, Zhengwen Liu

**Affiliations:** 1 Department of Infectious Diseases, First Affiliated Hospital of Xi’an Jiaotong University, Xi’an, Shaanxi, People’s Republic of China; 2 Department of Rehabilitation, First Affiliated Hospital of Xi’an Jiaotong University, Xi’an, Shaanxi, People’s Republic of China; Hong Kong Baptist University, HONG KONG

## Abstract

5-Hydroxytryptamine and its receptor are involved in hepatocarcinogenesis. This study investigated the effects of 5-hydroxytryptamine 3 receptor antagonists (5-HT3RAs) on the prognosis of patients with advanced hepatocellular carcinoma (HCC). Patients with advanced HCC who received best supportive care were analyzed. Overall survival (OS) between 5-HT3RA users and nonusers was compared. Propensity score matching (PSM) was used to balance variables. Factors associated with prognosis were identified by Cox regression. A total of 1141 patients with advanced HCC were included and 102 of them received 5-HT3RAs. 5-HT3RA treatment was significantly associated with reduced mortality [hazard ratio (HR) = 0.690; 95% confidence interval (CI): 0.546–0.871; *p* = 0.002]. After 1:2 PSM (n = 89 and n = 161 in 5-HT3RA user and nonuser groups, respectively), 5-HT3RA treatment remained significantly associated with reduced mortality (HR = 0.615; 95% CI: 0.457–0.829; *p* = 0.001). Subgroup analysis of hepatitis B virus-associated HCC patients yielded similar findings. Subgroup analysis of 5-HT3RAs also yielded similar findings, showing that the 5-year OS was significantly higher in tropisetron users than nonusers (23.7% *vs.* 10.9%, *p* = 0.004) after PSM. The use of ondansetron (HR = 0.603; 95% CI: 0.365–0.995; *p* = 0.048) or tropisetron (HR = 0.477; 95% CI: 0.299–0.759; *p* = 0.002) was independent factor associated with improved prognosis after PSM. Taken together, this study shows that the use of 5-HT3RAs is associated with improved OS and the use of ondansetron or tropisetron was independently associated with a better prognosis in patients with advanced HCC, indicating a favorable effect of 5-HT3RAs on HCC patients.

## Introduction

Primary liver cancer ranks as the sixth most commonly diagnosed cancer and the third leading cause of cancer mortality around the world in 2022 [1]. In China, it was the fourth most common cancer and the second leading cause of cancer-related deaths as of 2022 [2]. Hepatocellular carcinoma (HCC) is the most common type of primary liver cancer, comprising 75%−85% of cases [1]. Outcomes of HCC are poor mainly because many cases are diagnosed at an advanced stage. Patients with advanced HCC have limited treatment options and an extremely poor prognosis, primarily due to high tumor burden, a severe degree of liver dysfunction, and poor performance status [3]. Novel and alternative treatments besides best supportive care (BSC) are urgently needed for advanced HCC.

5-Hydroxytryptamine (5-HT, serotonin), a monoamine neurotransmitter in the central nervous system and an endogenous 5-HT receptor (5-HTR) agonist, plays regulatory roles in the nervous system, digestive system and cardiovascular system. 5-HTRs are classified into seven distinct subtypes and they are generally G protein-coupled receptors, except for the 5-HT3 receptors (5-HT3Rs), which are the only cation-conducting pentameric ligand-gated ion channels [4,5]. 5-HT regulates vomiting in the peripheral and central nervous system by binding to 5-HTRs. The 5-HT3R antagonists (5-HT3RAs) are widely used for the prevention and treatment of nausea and vomiting induced by chemotherapy, radiotherapy, anesthesia, and surgical procedures [6]. Noteworthyly, 5-HT and 5-HTRs are implicated in certain malignancies, including breast cancer [7], colorectal carcinoma [8], prostate cancer [9], bladder cancer [10], lung adenocarcinoma [11] and pancreatic tumor [12]. Furthermore, downregulation of 5-HTR3A expression can exert an anticancer activity against colorectal carcinoma cells *in vitro* [8]. Antagonism of 5HT could significantly inhibit bladder cancer cell growth [10]. 5-HT3RAs, palonosetron and ramosetron, have anti-tumor potential against lung cancer cells [13]. 5-HT3RA tropisetron has potential anti-tumor effect on lung adenocarcinoma [11] and pancreatic cancer [14].

As far as HCC is concerned, 5-HT has been revealed to promote HCC proliferation, invasion and metastasis [15–18] and contributes to the progression and poor prognosis of HCC [19]. 5-HT2AR has been shown to be a potential target for HCC immunotherapy [20]. Although 5-HT and 5-HTRs are involved in HCC, the effects of 5-HT3RAs, which are clinically used for the treatment of nausea and vomiting in patients with cancers including HCC, on the outcomes of patients with HCC remain unclear. Therefore, this study aims to evaluate the association between 5-HT3RA treatment and the prognosis of patients with advanced HCC undergoing BSC.

## Patients and methods

### Study population

Data from patients diagnosed with advanced HCC who underwent BSC at the First Affiliated Hospital of Xi’an Jiaotong University between June 2011 and July 2021 were retrospectively collected. The inclusion criteria for the patients comprised individuals aged over 18 years, meeting the diagnosis standards of “AASLD Practice Guidance on prevention, diagnosis, and treatment of HCC” [3], and being classified as advanced HCC, defined as Barcelona Clinic Liver Cancer (BCLC) stage C or D, and receiving BSC as the primary treatment. BCLC staging was performed based on the ECOG Performance Status, tumor burden including tumor number, tumor size and metastatic status, and liver function including Child-Pugh stage. BCLC stage was used as an integrated measure of HCC disease severity in this study. The majority of HCC in the patients (79.3%, 905 patients) were classified as BCLC stage D. Although systemic anti-tumor therapy is recommended by current guidelines for patients with BCLC stage C HCC, this study included a small proportion of stage C patients (20.7%) who received only BSC. This deviation from guideline-recommended treatment might be attributed to restricted drug availability, and socioeconomic factors, such as financial burden and educational level. Anyway, the choice of BSC in the stage C patients was also made with shared decision-making between physicians and patients. HCC diagnosis was based on the presence of arterial phase hyperenhancement and washout in the portal venous or delayed phases on contrast-enhanced multiphase computed tomography or magnetic resonance imaging [3]. The exclusion criteria were: (1) cancers other than HCC; (2) the presence of respiratory, circulatory, or renal failure; (3) loss to follow-up.

The study protocol conforms to the ethical guidelines of the Declaration of Helsinki. The Ethics Committee of the First Affiliated Hospital of Xi’an Jiaotong University approved the study (XJTU1AF2021LSK-307) and the need for informed consent was waived in view of the retrospective nature of the study and the anonymous data collection of the patients.

### Data collection

The anonymized demographic data were collected from the Biobank of the First Affiliated Hospital of Xi’an Jiaotong University. Demographic and clinical data and the treatments in the patients were collected from the electronic medical records. All the data accessed for analysis were collected between 27/09/2021 and 26/02/2025. The data included age, sex, body mass index (BMI), smoking, alcohol consumption, blood cell count, biochemical liver function and α-fetoprotein (AFP) levels. The etiology of the underlying liver disease, comorbidities such as hypertension and diabetes mellitus, nausea and vomiting symptoms, and complications of liver disease [ascites, spontaneous bacterial peritonitis (SBP), gastrointestinal bleeding due to esophageal and gastric varices, and hepatic encephalopathy (HE)] were also recorded. In patients with hepatitis B virus (HBV)-related HCC, hepatitis B surface antigen (HBsAg) and hepatitis B e antigen (HBeAg) status were recorded. Treatments including the use of antivirals in HBV infected patients and the use of 5-HT3RAs in addition to BSC were recorded.

### Calculation of INVR, FIB-4 and mALBI in the patients

Nausea and vomiting at baseline were assessed using the Index of Nausea, Vomiting, and Retching (INVR) [21]. Hepatic fibrosis was evaluated with the fibrosis-4 index (FIB-4) [22,23]. Modified Albumin-Bilirubin (mALBI) grade was computed based on serum albumin and total-bilirubin values as previously described [24].

### 5-HT3RA exposure and outcome

5-HT3RAs were administered in patients with advanced HCC experiencing nausea and/or vomiting that required symptomatic treatment, at the discretion of clinicians. 5-HT3RAs used included ondansetron, tropisetron, palonosetron and granisetron. The primary outcome of this study was overall survival (OS), defined as the time from study inclusion to death from any cause.

### Statistical analysis

Categorical variables were described as frequencies (percentages) and were compared using the Pearson chi-square analysis or Fisher’s exact test. Continuous variables were tested for normality. The normally distributed variables were described using mean ± standard deviation and were compared using the Student's t-test. The non-normally distributed variables were presented as the median (interquartile range, IQR) and were compared using the Mann-Whitney *U* test or Kruskal–Wallis test. The OS was estimated by using the Kaplan-Meier method and the log-rank test. In addition, collinearity between variables was detected by calculating the correlation coefficient and the *p-value* between the variables. It was generally accepted that a correlation coefficient greater than 0.7 combined with a *p-value* less than 0.05 may indicate potential collinearity among variables. Two commonly used metrics for diagnosing multicollinearity, namely tolerance and the variance inflation factor (VIF), suggest the presence of multicollinearity when tolerance is below 0.2 or VIF exceeds 10. In the present study, all pairwise correlation coefficients were below 0.7, tolerance values were above 0.2, and VIFs were less than 10. These results indicate that no multicollinearity existed among the variables examined. The Cox proportional-hazards regression model was used to assess the impact of all baseline covariates on the outcomes. In the regression model, hazard ratios (HRs) and 95% confidence intervals (CIs) were calculated for each variable. Propensity score matching (PSM) was used to minimize the influence of data bias and confounding variables on the outcome. After calculating the propensity score, we used the 5-HT3RA user group and the 5-HT3RA nonuser group for patients with similar propensity scores using a 1:2 ratio matching. All statistical analyses were conducted using R version 4.4.3 for Mac (The R Foundation for Statistical Computing, Vienna, Austria). According to the test level of α = 0.05, a two-sided *p-value* < 0.05 was considered statistically significant.

## Results

### Patient characteristics

During the study period, a total of 1208 patients were diagnosed with advanced HCC undergoing BSC in the First Affiliated Hospital of Xi’an Jiaotong University. Of these patients, 46 patients were excluded based on predefined criteria (25 with other malignant tumors and 21 with respiratory failure, circulatory failure, or renal failure) and 21 patients were lost to follow-up. Finally, 1141 patients were deemed eligible for the inclusion in the study (Fig 1). The patients were divided into 5-HT3RA user (n = 102) and 5-HT3RA nonuser (n = 1039, Fig 1) groups according to the administration of 5-HT3RAs. PSM was performed at a 1:2 ratio to match all characteristics and 250 patients were selected (89 patients in 5-HT3RA user group and 161 patients in 5-HT3RA nonuser group, Fig 1).

**Fig 1:**
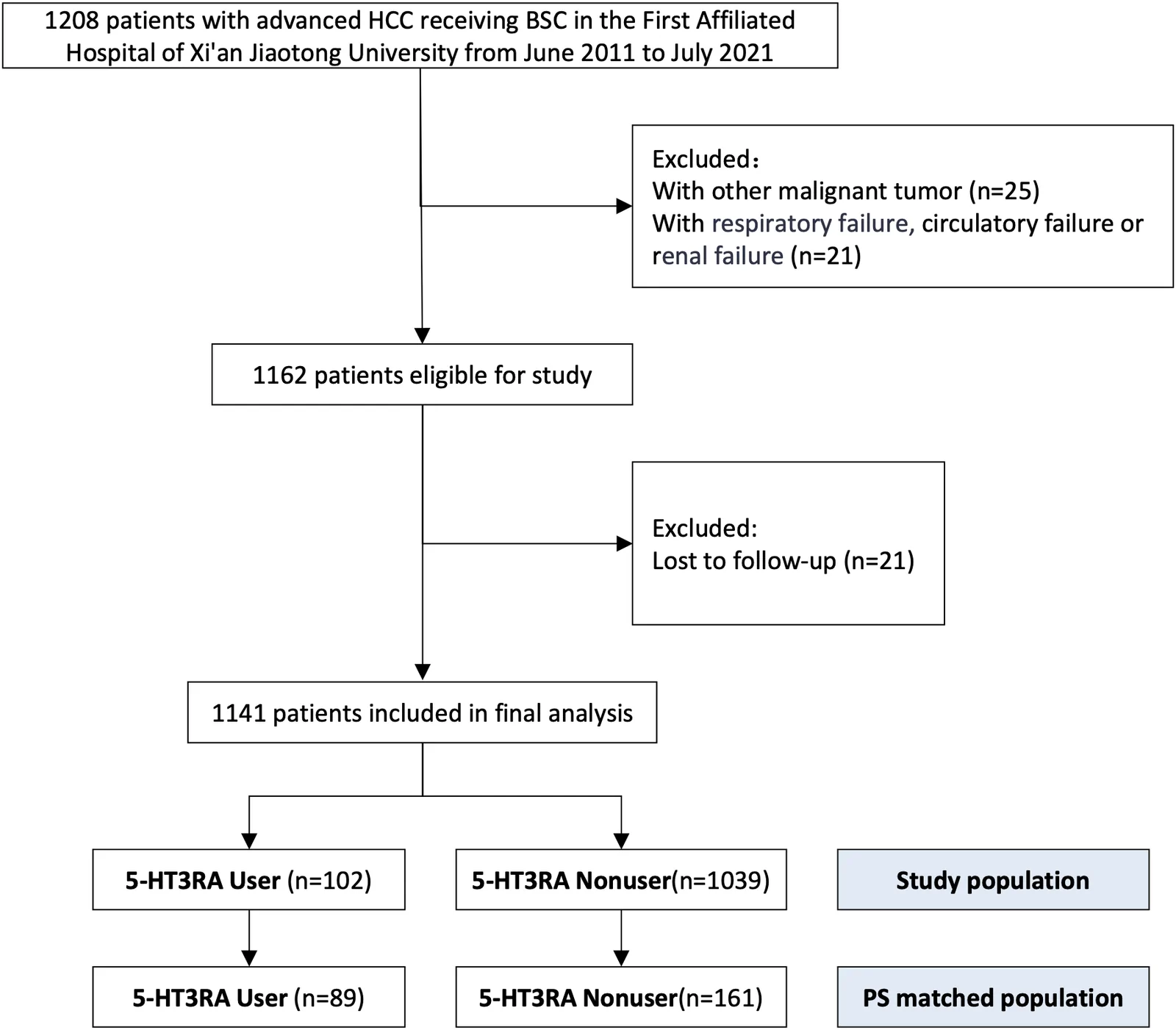
Flow chart showing the selection of the study population. BSC, best supportive care; HCC, hepatocellular carcinoma; PS, propensity score.

The baseline characteristics of the patients are summarized in Table S1 in S1 File. Among the study population, the proportion of males was higher (75.2% and 79.7% in the 5-HT3RA user and 5-HT3RA nonuser groups, respectively). HBV was the most common etiology (79.4% and 83.1% in the 5-HT3RA user and 5-HT3RA nonuser groups, respectively). The majority of patients were classified as BCLC stage D, comprising 79.3% (n = 905) of the overall population, with 77.5% (n = 79) and 79.5% (n = 826) patients in the 5-HT3RA user and nonuser groups, respectively. Compared with 5-HT3RA nonuser group, patients in 5-HT3RA user group were significantly more often diagnosed with SBP and had a higher mALBI grade. After PSM, the baseline characteristics between the two groups were not statistically different (see Table S2 in S1 File).

### Overall survival analysis

Death was observed in 951 patients in the overall cohort, including 79 (77.5%) patients in 5-HT3RA user group and 872 (85.9%) patients in 5-HT3RA nonuser group. After PSM, 209 deaths were recorded, with 71 (79.8%) in the 5-HT3RA user group and 138 (85.7%) in the nonuser group, respectively. The 5-year OS was significantly higher in 5-HT3RA user group than in 5-HT3RA nonuser group both before PSM (20.0% *vs*. 13.3%, *p* = 0.031, Fig 2A) and after PSM (18.3% *vs*. 12.9%, *p* = 0.015, Fig 2B).

**Fig 2:**
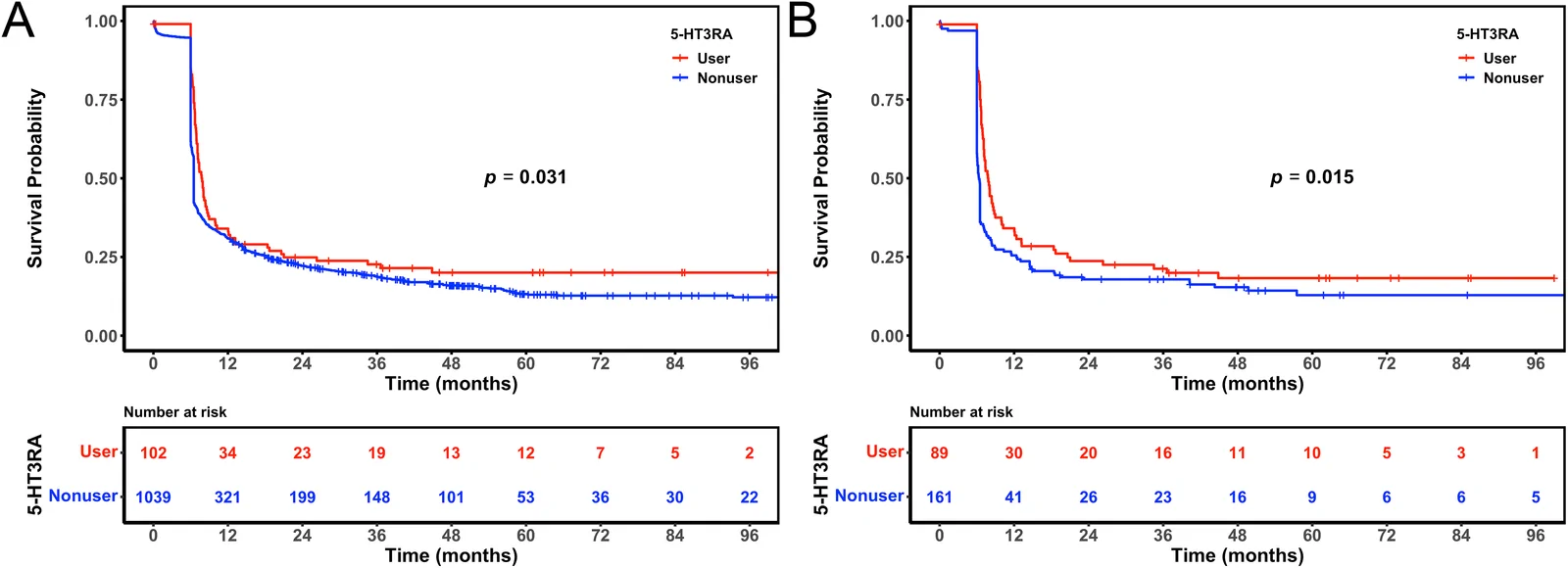
Kaplan-Meier curves of overall survival (OS) in patients with and without the use of 5-hydroxytryptamine 3 receptor antagonists (5-HT3RAs). A. OS in the entire study population; B. OS in the propensity score-matched population.

### Factors associated with prognosis of patients

Cox regression analyses revealed that before PSM, the use of 5-HT3RAs was significantly related to the prognosis of patients (HR = 0.690; 95% CI: 0.546–0.871; *p* = 0.002, see Table S3 in S1 File). In addition, mALBI grade 2b (HR = 1.261; 95% CI: 1.014–1.568; *p* = 0.037), grade 3 (HR = 1.330; 95% CI: 1.030–1.716; *p* = 0.029), BCLC stage D (HR = 1.579; 95% CI: 1.333–1.871; *p* < 0.001) and AFP ≥ 400 ng/mL (HR = 1.207; 95% CI: 1.059–1.376; *p* = 0.005) were also significantly related to the prognosis of patients (see Table S3 in S1 File). After PSM, multivariate analysis showed that the use of 5-HT3RAs (HR = 0.615; 95% CI: 0.457–0.829; *p* = 0.001), in addition to mALBI grade 3 (HR = 2.222; 95% CI: 1.233–4.007; *p* = 0.008), was independently associated with the prognosis of patients (Table 1).

**Table 1:** Univariate and multivariate Cox regression analyses of variables and overall survival in the patients after propensity score matching.

	Univariate			Multivariate		
	HR	95% CI	*p* value	HR	95% CI	*p* value
Sex						
Female	Reference			Reference		
Male	0.995	0.731-1.354	0.973	1.007	0.719-1.411	0.965
Hypertension	1.165	0.803-1.690	0.422			
Diabetes mellitus	1.140	0.684-1.899	0.615			
Smoking	1.051	0.799-1.384	0.720			
Alcohol use	1.032	0.728-1.462	0.861			
Family history of HCC	1.249	0.712-2.191	0.437			
Complication						
Ascites	1.078	0.807-1.439	0.613	0.824	0.590-1.151	0.256
SBP	1.569	1.090-2.260	0.015	1.440	0.929-2.234	0.103
HE	1.305	0.643-2.647	0.461	0.612	0.278-1.345	0.222
Variceal bleeding	1.367	0.820-2.279	0.230	1.071	0.603-1.903	0.814
FIB-4						
F0-F2	Reference			Reference		
F3, F4	0.916	0.450-1.867	0.810	0.756	0.354-1.616	0.471
mALBI grade						
1	Reference			Reference		
2a	1.190	0.629-2.252	0.592	1.106	0.579-2.116	0.760
2b	1.600	0.971-2.638	0.065	1.645	0.974-2.778	0.063
3	2.235	1.319-3.788	0.003	2.222	1.233-4.007	0.008
BCLC stage						
C	Reference			Reference		
D	1.415	1.009-1.983	0.044	1.260	0.876-1.813	0.213
AFP (ng/mL)						
< 400	Reference			Reference		
≥ 400	1.020	0.775-1.343	0.886	1.069	0.793-1.442	0.660
5-HT3RAs						
Nonuser	Reference			Reference		
User	0.671	0.503-0.895	0.007	0.615	0.457-0.829	0.001

Abbreviations: HCC, hepatocellular carcinoma; HR, hazard ratio; CI, confidence interval; SBP, spontaneous bacterial peritonitis; HE, hepatic encephalopathy; FIB-4, fibrosis-4 index; mALBI, modified albumin-bilirubin grade; BCLC, Barcelona Clinic Liver Cancer; AFP, α-fetoprotein; 5-HT3RA, 5-HT3 receptor antagonist.

### Subgroup analysis

Etiologically, since the most common etiology of HCC in the patients included was HBV (79.4% and 83.1% in the 5-HT3RA user and 5-HT3RA nonuser groups, respectively), patients with HBV-related HCC were analyzed. Of the 944 patients with HBV-related HCC, 81 received 5-HT3RAs treatment. The baseline characteristics of the HBV-related HCC patients are summarized in Table S4 in S1 File. Significant differences were observed between the two groups in the proportion of male patients, the prevalence of SBP, INVR total score, mALBI grade, and the use of adefovir before PSM (see Table S4 in S1 File). After 1:2 PSM, 80 patients in the 5-HT3RAs user group and 158 patients in the 5-HT3RAs nonuser group were included, and the baseline characteristics were comparable between the two groups (see Table S5 in S1 File).

In patients with HBV-related HCC, the 5-year OS in 5-HT3RA user group was significantly higher than that in 5-HT3RAs nonuser group both before (22.6% *vs.* 13.1%, *p* = 0.009, Fig 3A) and after (22.9% *vs.* 12.0%, *p* = 0.004, Fig 3B) PSM.

**Fig 3:**
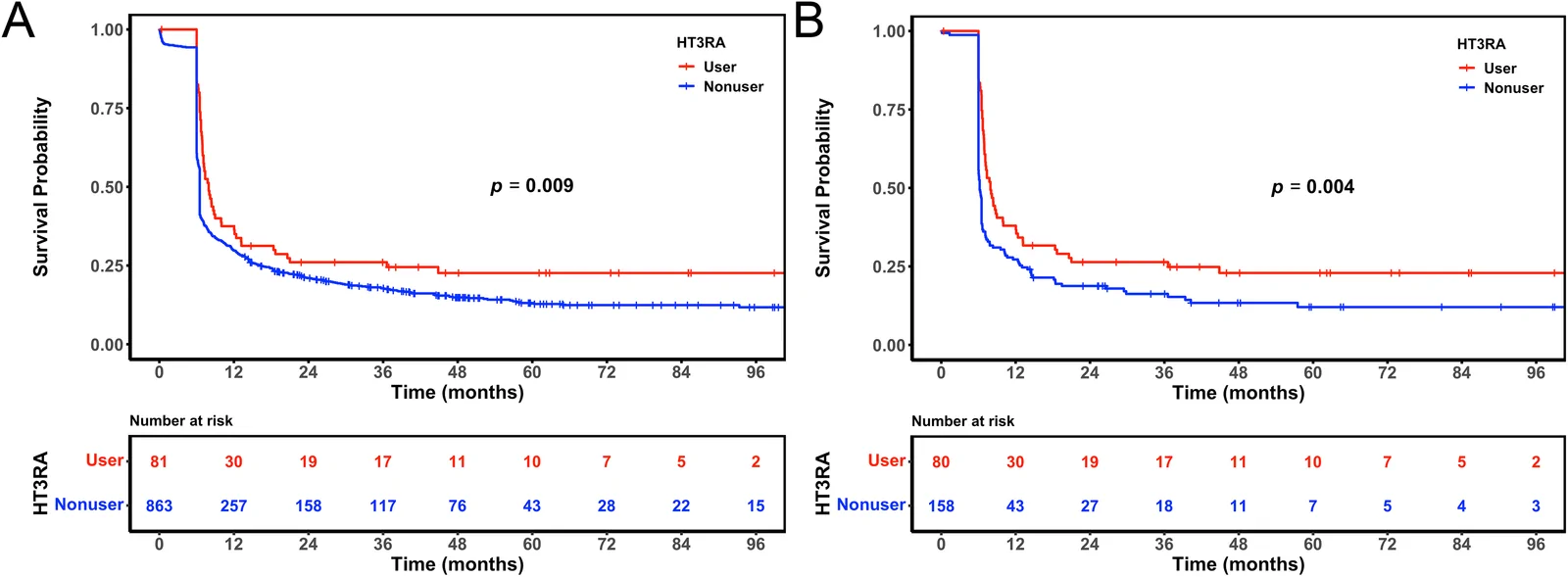
Kaplan-Meier curves of overall survival (OS) in hepatitis B virus-related hepatocellular carcinoma (HCC) patients with and without the use of 5-hydroxytryptamine 3 receptor antagonists (5-HT3RAs). A. OS in the overall population with HBV-related HCC; B. OS in the propensity score-matched population with HBV-related HCC.

Multivariate analysis before PSM identified that the use of 5-HT3RAs was associated with the prognosis of patients (HR = 0.611; 95% CI: 0.469–0.797; *p* < 0.001, see Table S6 in S1 File). In addition, mALBI grade 2b (HR = 1.312; 95% CI: 1.040–1.656; *p* = 0.022) and grade 3 (HR = 1.427; 95% CI: 1.081–1.883; *p* = 0.012), BCLC stage D (HR = 1.599; 95% CI: 1.331–1.920; *p* < 0.001), AFP ≥ 400 ng/mL (HR = 1.219; 95% CI: 1.056–1.407; *p* = 0.007), and the use of antiviral agents (HR = 0.809; 95% CI: 0.701–0.935; *p* = 0.004) were associated with the prognosis of patients. Among the antiviral agents, the use of entecavir (HR = 0.803; 95% CI: 0.693–0.930; *p* = 0.004) was an independent factor associated with the OS (see Table S6 in S1 File). After PSM, multivariate analysis revealed that the use of 5-HT3RAs (HR = 0.589; 95% CI: 0.430–0.807; *p* = 0.001) was significantly associated with the prognosis of the patients. In addition, BCLC stage D (HR = 1.505; 95% CI: 1.028–2.204; *p* = 0.035) and the use of entecavir (HR = 0.712; 95% CI: 0. 0.524–0.969; *p* = 0.030) were also significant prognostic factors (Table 2).

**Table 2:** Univariate and multivariate Cox regression analyses of variables and overall survival in HBV-related hepatocellular carcinoma patients after propensity score matching.

	Univariate			Multivariate		
	HR	95% CI	*p* value	HR	95% CI	*p* value
Sex						
Female	Reference			Reference		
Male	1.012	0.727-1.409	0.944	1.060	0.741-1.515	0.751
Complication						
Ascites	0.853	0.636-1.142	0.285	0.740	0.530-1.034	0.078
SBP	1.332	0.920-1.929	0.129	1.519	0.985-2.343	0.059
HE	2.468	0.609-9.992	0.206	1.732	0.367-8.185	0.488
Variceal bleeding	1.041	0.641-1.690	0.872	0.838	0.463-1.516	0.559
FIB-4						
F0-F2	Reference			Reference		
F3, F4	0.740	0.183-2.991	0.673	0.507	0.117-2.191	0.363
mALBI grade						
1	Reference			Reference		
2a	0.758	0.327-1.756	0.517	0.743	0.314-1.757	0.499
2b	1.327	0.733-2.402	0.351	1.239	0.678-2.265	0.487
3	1.265	0.675-2.372	0.463	1.214	0.632-2.333	0.561
BCLC stage						
C	Reference			Reference		
D	1.934	0.944-1.934	0.100	1.505	1.028-2.204	0.035
AFP (ng/mL)						
< 400	Reference			Reference		
≥ 400	1.350	0.77-1.35	0.894			
HBeAg positive	0.717	0.488-1.053	0.090	0.699	0.465-1.050	0.084
Antiviral agent	0.852	0.643-1.128	0.263	0.804	0.593-1.089	0.159
Entecavir (ETV)	0.771	0.580-1.023	0.072	0.712	0.524-0.969	0.030
Tenofovir alafenamide (TAF)	0.353	0.049-2.523	0.300	0.214	0.025-1.818	0.158
Tenofovir disoproxil fumarate (TDF)	1.408	0.579-3.425	0.451	1.518	0.597-3.858	0.381
Adefovir (ADV)	1.121	0.552-2.276	0.752	1.550	0.588-4.083	0.375
Lamivudine (LAM)	1.122	0.553-2.277	0.750	0.912	0.392-2.125	0.832
5-HT3RA						
Nonuser	Reference			Reference		
User	0.617	0.455-0.838	0.002	0.589	0.430-0.807	0.001

Abbreviations: HBV, hepatitis B virus; HCC, hepatocellular carcinoma; HR, hazard ratio; CI, confidence interval; SBP, spontaneous bacterial peritonitis; HE, hepatic encephalopathy; FIB-4, fibrosis-4 index; mALBI, modified albumin–bilirubin grade; BCLC, Barcelona Clinic Liver Cancer; AFP, α-fetoprotein; ETV, entecavir; TAF, tenofovir alafenamide; TDF, tenofovir disoproxil fumarate; ADV, adefovir; LAM, lamivudine; 5-HT3RA, 5-HT3 receptor antagonist.

In relation to different 5-HT3RAs, since the most commonly used 5-HT3RAs were ondansetron and tropisetron, their associations with patient prognosis were respectively analyzed.

In terms of ondansetron, compared with ondansetron nonusers (n = 1039), ondansetron users (n = 47) had higher SBP (27.7% *vs*. 9.8%, *p* < 0.001), INVR total score (12.00 *vs.* 6.00, *p* < 0.001) and mALBI grade (*p* = 0.009, see Table S7 in S1 File) before PSM. After PSM, the characteristics and laboratory data in patients with (n = 37) and without (n = 71) the use of ondansetron had no significant differences (see Table S8 in S1 File).

The 5-year OS did not differ significantly between ondansetron user and nonuser groups, either before [18.3% *vs*. 13.3%, *p* = 0.29, Fig 4A (a)] or after [15.4% *vs*. 15.3%, *p* = 0.54, Fig 4A (b)] PSM.

**Fig 4:**
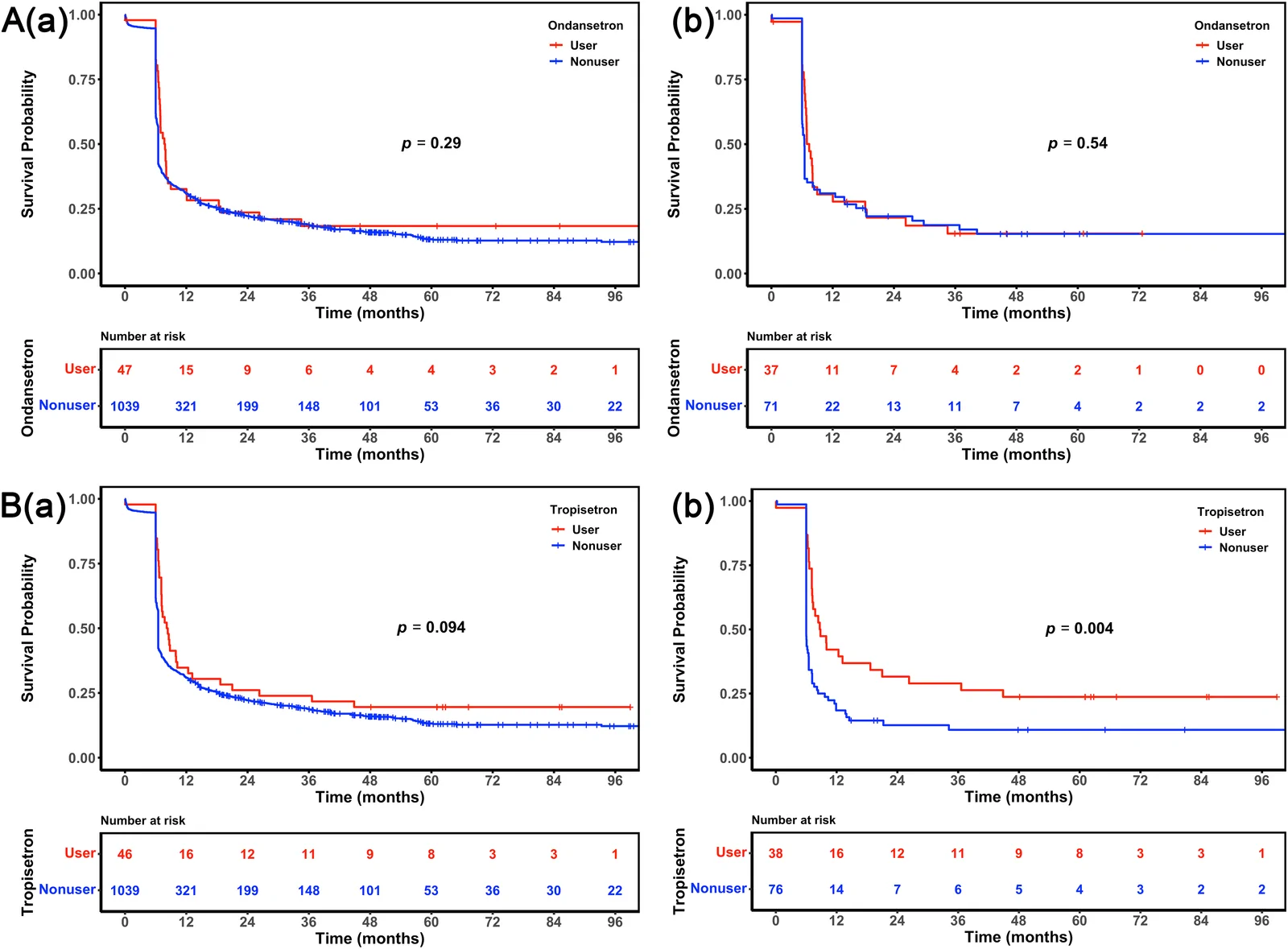
Kaplan–Meier curves of overall survival (OS) in patients with and without the use of different 5-hydroxytryptamine 3 receptor antagonists. A. OS in patients with and without the use of ondansetron. (a) before propensity score-matching (PSM). (b). after PSM; B. OS in patients with and without the use of tropisetron. (a) before PSM. (b). after PSM.

Before PSM, univariate analyses showed that ascites (*p* = 0.001), SBP (*p* = 0.006), FIB-4 (*p* = 0.009) and mALBI grade (*p* < 0.001 for both grade 2b and grade 3) were associated with the prognosis of patients (see Table S9 in S1 File). Multivariate analyses showed that FIB-4 grade F3 or F4 (HR = 1.346; 95% CI: 1.003–1.807; *p* = 0.048), mALBI grade 2b (HR = 1.264; 95% CI: 1.011–1.580; *p* = 0.040) and grade 3 (HR = 1.324; 95% CI: 1.015–1.727; *p* = 0.038), BCLC stage D (HR = 1.575; 95% CI: 1.323–1.875; *p* < 0.001) and AFP ≥ 400 ng/mL (HR = 1.231; 95% CI: 1.075–1.410; *p* = 0.003) were associated with the prognosis of patients (see Table S9 in S1 File). The use of ondansetron was associated with a borderline significant trend toward improved survival (HR = 0.712; 95% CI: 0.507–1.000; *p* = 0.050, see Table S9 in S1 File). After PSM, multivariate analyses showed that, in addition to smoking (HR = 2.015; 95% CI: 1.071–3.791; *p* = 0.030) and BCLC stage D (HR = 1.928; 95% CI: 1.104–3.369; *p* = 0.021), the use of ondansetron was independently associated with the prognosis of patients (HR = 0.603; 95% CI: 0.365–0.995; *p* = 0.048, Table 3).

**Table 3:** Univariate and multivariate Cox regression analyses of variables and overall survival in patients with and without the use of ondansetron after propensity score matching.

	Univariate			Multivariate		
	HR	95% CI	*p* value	HR	95% CI	*p* value
Sex						
Female	Reference			Reference		
Male	1.403	0.887-2.221	0.148	1.184	0.624-2.245	0.605
Hypertension	1.255	0.739-2.131	0.400			
Diabetes mellitus	0.947	0.412-2.172	0.897			
Smoking	1.432	0.935-2.195	0.099	2.015	1.071-3.791	0.030
Alcohol use	1.688	0.934-3.048	0.083	1.305	0.634-2.687	0.469
Family history of HCC	1.039	0.537-2.008	0.910			
Complication						
Ascites	0.749	0.487-1.154	0.190			
SBP	1.262	0.767-2.079	0.360			
HE	1.295	0.179-9.361	0.798			
Variceal bleeding	0.956	0.520-1.758	0.885			
FIB-4						
F0-F2	Reference			Reference		
F3, F4	0.134	0.040-0.444	0.001	0.594	0.349-1.009	0.054
mALBI grade						
1	Reference			Reference		
2a	1.024	0.371-2.827	0.964	1.239	0.355-4.326	0.737
2b	1.021	0.459-2.271	0.959	1.143	0.380-3.434	0.812
3	1.138	0.499-2.595	0.759	1.629	0.517-5.134	0.405
BCLC stage						
C	Reference			Reference		
D	1.588	0.977-2.581	0.062	1.928	1.104-3.369	0.021
AFP (ng/mL)						
< 400	Reference			Reference		
≥ 400	1.214	0.793-1.859	0.372	1.415	0.824-2.431	0.208
Ondansetron						
Nonuser	Reference			Reference		
User	0.825	0.530-1.283	0.393	0.603	0.365-0.995	0.048

Abbreviations: HCC, hepatocellular carcinoma; HR, hazard ratio; CI, confidence interval; SBP, spontaneous bacterial peritonitis; HE, hepatic encephalopathy; FIB-4, fibrosis-4 index; mALBI, modified albumin–bilirubin grade; BCLC, Barcelona Clinic Liver Cancer; AFP, α-fetoprotein.

Regarding tropisetron, compared with tropisetron nonuser group (n = 1039), tropisetron user group (n = 46) had higher INVR total score (11.00 *vs.* 6.00, *p* < 0.001, see Table S10 in S1 File) before PSM. After 1:2 PSM, baseline characteristics and laboratory parameters were comparable between tropisetron users (n = 38) and nonusers (n = 76; see Table S11 in S1 File).

Before PSM, the 5-year OS was higher in tropisetron users than in nonusers, though not statistically significant [19.6% *vs*. 13.3%, *p* = 0.094, Fig 4B (a)]. After PSM, the difference became statistically significant [23.7% *vs*. 10.9%, *p* = 0.004, Fig 4B (b)].

Before PSM, univariate analyses showed that ascites (*p* = 0.001), SBP (*p* = 0.002), FIB-4 (*p* = 0.008), mALBI grade (*p* < 0.001 for both grade 2b and grade 3), BCLC stage D (*p* < 0.001) and AFP ≥ 400 ng/mL (*p* < 0.001) were associated with the prognosis of patients (see Table S12 in S1 File). Multivariate analyses showed that male gender (HR = 1.188; 95% CI: 1.006–1.403; *p* = 0.042), FIB-4 (HR = 1.354; 95% CI: 1.012–1.811; *p* = 0.041), mALBI grade 3 (HR = 1.343; 95% CI: 1.037–1.739; *p* = 0.026), BCLC stage D (HR = 1.589; 95% CI: 1.336–1.889; *p* < 0.001), and AFP ≥ 400 ng/mL (HR = 1.185; 95% CI: 1.037–1.355; *p* = 0.013) were associated with the prognosis of patients (see Table S12 in S1 File). Treatment with tropisetron demonstrated a borderline statistically significant trend toward improved survival (HR = 0.718; 95% CI: 0.516–1.000; *p* = 0.050, see Table S12 in S1 File). After PSM, univariate analyses showed that, in addition to family history of HCC (HR = 4.261; 95% CI: 1.025–17.715; *p* = 0.046), the use of tropisetron was associated with the prognosis of patients (HR = 0.501; 95% CI: 0.323–0.779; *p* = 0.002, Table 4). Multivariate analyses showed that only the use of tropisetron was independently associated with the prognosis of patients (HR = 0.477; 95% CI: 0.299–0.759; *p* = 0.002, Table 4).

**Table 4:** Univariate and multivariate Cox regression analyses of variables and overall survival in patients with and without the use of tropisetron after propensity score matching.

	Univariate			Multivariate		
	HR	95% CI	*p* value	HR	95% CI	*p* value
Sex						
Female	Reference			Reference		
Male	1.047	0.667-1.643	0.842	0.971	0.609-1.548	0.901
Hypertension	1.726	0.912-3.266	0.094			
Diabetes mellitus	0.652	0.285-1.492	0.311			
Smoking	1.205	0.800-1.816	0.372			
Alcohol use	0.918	0.536-1.572	0.755			
Family history of HCC	4.261	1.025-17.715	0.046	2.190	0.414-11.58	0.356
Complication						
Ascites	0.947	0.600-1.494	0.815	0.774	0.446-1.343	0.362
SBP	1.616	0.592-4.415	0.349	1.399	0.325-6.012	0.652
HE	0.983	0.242-3.998	0.981	0.415	0.063-2.72	0.360
Variceal bleeding	1.152	0.160-8.308	0.888	2.649	0.305-22.967	0.377
FIB-4						
F0-F2	Reference			Reference		
F3, F4	1.029	0.445-2.378	0.947	1.005	0.403-2.505	0.991
mALBI grade						
1	Reference			Reference		
2a	1.558	0.687-3.534	0.289	1.785	0.771-4.132	0.176
2b	1.445	0.749-2.788	0.273	1.326	0.667-2.639	0.421
3	1.734	0.850-3.535	0.130	1.393	0.612-3.169	0.429
BCLC stage						
C	Reference			Reference		
D	1.386	0.797-2.409	0.247	1.421	0.758-2.662	0.273
AFP (ng/mL)						
< 400	Reference			Reference		
≥ 400	0.998	0.667-1.493	0.992	0.993	0.630-1.566	0.977
Tropisetron						
Nonuser	Reference			Reference		
User	0.501	0.323-0.779	0.002	0.477	0.299-0.759	0.002

Abbreviations: HCC, hepatocellular carcinoma; HR, hazard ratio; CI, confidence interval; SBP, spontaneous bacterial peritonitis; HE, hepatic encephalopathy; FIB-4, fibrosis-4 index; mALBI, modified albumin–bilirubin grade; BCLC, Barcelona Clinic Liver Cancer; AFP, α-fetoprotein.

## Discussion

5-HTs and their receptors are involved in the carcinogenesis of certain cancers, including HCC [15–20]. 5-HT3RAs are widely used for the treatment of nausea and vomiting in cancer patients. However, the effect of 5-HT3RAs on the outcomes of patients with HCC remains unclear. This study showed that the use of 5-HT3RAs in patients with advanced HCC undergoing BSC was associated with prolonged OS. Furthermore, the use of 5-HT3RA tropisetron and ondansetron was independent factors associated with improved OS in patients with advanced HCC.

The underlying mechanisms associated with the survival benefit of 5-HT3RA use in advanced HCC patients remain to be investigated. The prognosis of patients with advanced HCC is determined by various factors. Patients’ performance, liver function and tumor condition as well as treatment regiments are all important determinants for the prognosis. The findings in the present study suggest the potential multiple benefits of 5-HT3RAs for advanced HCC patients in that only the OS in the patients was evaluated in relation to the usage of 5-HT3RAs.

On the one hand, 5-HT3RAs are revealed to have antiinflammatory effects in both animal and human. The antiphlogistic effects of the 5-HT3RA tropisetron are shown to be mediated by the inhibition of p38 mitogen-activated protein kinases (MAPK) activation [25] and the inhibition of tumor necrosis factor (TNF)-α/interleukin (IL)-1 β [26]. Moreover, 5-HT3RA tropisetron may protect against acetaminophen-induced liver injury through the suppression of hepatic oxidative stress and the modulation of the activation of c-jun N-terminal kinase (JNK)/extracellular signal-regulated kinase (ERK) MAPK pathways [27]. 5-HT3RA palonosetron could reduce cisplatin-induced cytotoxicity by inhibiting p38 phosphorylation [28]. In animals, 5-HT3RA tropisetron is revealed to be able to inhibit sepsis by suppressing hyper-inflammation and modulating the cardiac action potential [29] and to ameliorate D-galactose (DG)-induced heart [30], renal [31] and brain aging [32] via modulation of sirtuin 1 (SIRT1) signaling. Tropisetron can also attenuate cisplatin-induced nephrotoxicity in mice [33] and alleviate diabetes-induced peripheral nerve injury through its anti-inflammatory and anti-apoptotic effects by modulating TNF-α and IL-1β and Bax/Bcl-2 ratio in animals [34]. Clinically, the use of 5-HT3RAs has been shown to be associated with increased survival in patients with liver failure [35]. The 5-HT3RA ondansetron usage is shown to be associated with lower any cause mortality in COVID-19 [36]. 5-HT3RA tropisetron has been revealed to have cardioprotective and anti-inflammatory effects in patients undergoing heart valve replacement surgery with cardioplegic arrest [37]. 5-HT3RA ondansetron use before mechanical ventilation support was shown to be associated with a decreased risk of 30-day mortality in patients with acute respiratory distress syndrome [38]. The use of 5-HT3RAs could lower risk of cisplatin-induced acute kidney injury (AKI) in patients with cancer [39]. The 5-HT3RA palonosetron usage was associated with significantly lower incidence of cisplatin-induced nephrotoxicity in patients with esophageal cancer [40]. Therefore, it is suggested that the HCC patients may benefit from the antiinflammatory and hepatoprotective effects as well as the multi-organ protective effects of 5-HT3RAs.

On the other hand, studies have showed that 5-HT3RAs have anti-tumor effects in certain cancers. For instance, 5-HT3RAs ondansetron, palonosetron and ramosetron have anti-tumor effects in gastric cancer through G1 cell cycle regulation and immunomodulation [41]. *In vitro* experiments showed that 5-HT3RAs palonosetron and ramosetron had inhibitory effect on cell proliferation and colony formation and reduced migration in autophagic cell death in lung cancer cells via the ERK pathway [13]. Perioperative administration of 5-HT3RAs palonosetron and ramosetron was associated with lower recurrence rate after lung cancer surgery [13]. These findings suggest the anti-tumor potential of 5-HT3RAs palonosetron and ramosetron against lung cancer cells. 5-HT3RA tropisetron was also shown to have potential anti-tumor effect on lung adenocarcinoma [11]. Tropisetron could also inhibit pancreatic cancer [14]. With regard to HCC, in a study of 176 patients with HCC, 5-HT1B and 5-HT2B receptors were detected in 32% and 35% of HCC specimens, respectively, and their expression was associated with increased tumor proliferative activity. In HCC cell lines, serotonin stimulation promoted cell proliferation through activation of the MAPK/ERK pathway, whereas antagonism of 5-HT1B or 5-HT2B reduced cell proliferation [42]. Although the protein expression and functional significance in HCC have not been adequately validated, *HTR3A* has been reported among genes dysregulated in HCC in bioinformatic analyses [43]. The downregulation of 5-HTR3A expression has been shown to be able to induce an anticancer effect against colorectal carcinoma cells *in vitro* [8]. Therefore, whether the antagonism of HTR3A by 5-HT3RAs might also induce a potential anticancer effect on HCC remains to be studied. Previous study has also shown that the ERK/JNK/p38 MAPK signaling pathway is involved in hepatocarcinogenesis [44]. 5-HT3RA tropisetron is able to inhibit p38 MAPK activation [25] and modulate the activation of JNK/ERK MAPK pathways [27]. Therefore, it is also indicated that 5-HT3RAs might have potential anti-cancer effects against HCC through the regulation of MAPK-related pathways. The HCC patients may also benefit from the anticancer effects of 5-HT3RAs.

Tropisetron appeared to show a greater survival benefit than ondansetron in our study. Mechanistic difference of actions may relate to the different effects on HCC between tropisetron and ondansetron. Although both agents are potent 5-HT3RAs, tropisetron has additional agonistic effect on α-7 nicotinic acetylcholine receptors (α7nAChRs). The activation of α7nAChR could inhibit inflammatory and apoptotic signaling pathways in the oxidative stress conditions. For instance, tropisetron could ameliorate cyclophosphamide-induced bladder hemorrhage and inflammation in rats in a α7nAChR dependent manner [45]. The hepatic-protective effect of remifentanil in hepatic ischemia/reperfusion injury was shown to be through upregulating α7nACh to inhibit inflammation mediated by NF-κB activation [46]. The protective effect of menthol on liver and brain was indicated to be mediated by suppressing oxidative stress and inflammation, augmenting expression of brain-derived neurotrophic factor and α7nAChR [47]. Interestingly, a recent study showed that tropisetron could exert both antifibrotic and anti-inflammatory effects via α7nAChR-mediated signaling and enhanced excessive extracellular matrix remodeling in keloid fibroblasts and a rat incisional scar model [48]. It is suggested that the agonistic action to α7nAChRs may partly responsible for the difference of tropisetron from ondansetron.

The results from the present study will provide implication for the reference of clinical practice. 5-HT3RAs are extensively used as efficacious agents in counteracting nausea and emesis induced by various reasons such as chemotherapy. The potential benefits of 5-HT3RAs including ondansetron and tropisetron might be preferably considered when selecting antiemetics in patients with HCC. The various favorable effects of 5-HT3RAs based on multiple mechanisms also render 5-HT3RAs, including ondansetron and tropisetron, many re-purposing potentials for different clinical conditions.

Our study has some limitations. Firstly, this is a retrospective analysis performed at a single institution and uncontrolled and unrecognized biases are frequent in such studies. Therefore, potential selection bias could not be completely avoided. To reduce potential selection bias, patients were enrolled in strict accordance with inclusion and exclusion criteria, and PSM was undertaken to balance baseline characteristics. In addition, the retrospective design allows a real world study. Secondly, the sample size, especially in the subgroup analysis, is relatively small and this can limit statistical power. Thirdly, we only compared the OS in the patients. Tumor response assessments are not performed and this limits the provision of information regarding the potential effect of 5-HT3RAs on tumor. Also, a cause-specific mortality was not evaluated in our study because it is sometimes difficult to reliably distinguish liver-related deaths from non-liver-related deaths in these patients with advanced disease, especially in those with multiple disease conditions such as multiorgan failure. Therefore, this study aimed to explore the association between 5-HT3RAs and OS in patients with advanced HCC. Fourthly, the dose-dependent effect of 5-HT3RAs was not examined and the details regarding the duration, cumulative dose, and frequency of 5-HT3RA administration were not provided owing to the relatively small number of patients receiving 5-HT3RAs and the variations in 5-HT3RA administrations when categorization of subgroups was performed according to doses, duration and frequency with different 5-HT3RAs. Of course, clarification for these issues is required in future studies. In addition, inflammatory biomarkers were not included and analyzed in our study because these biomarkers were not routinely measured in HCC patients. Further studies correlating inflammatory biomarkers with 5-HT3RA treatment or survival outcomes are warranted. Altogether, all these shortcomings need to be addressed in future studies.

### Conclusions

This study found a significant association between the use of 5-HT3RAs and a better OS in patients with advanced HCC who underwent BSC and showed that the use of 5-HT3RAs ondansetron or tropisetron was independently associated with improved prognosis of the patients, suggesting that the use of 5-HT3RAs is a favourable prognostic factor for patients with advanced HCC. Further well-designed prospective studies with larger sample sizes are warranted to validate these findings and inform for the favorable management of HCC patients.

## Supporting information

S1 File Supplementary data Tables S1–S12. (DOCX)

S2 File Baseline data used for the baseline characteristics analysis and PSM. (XLS)

S3 File Patient outcome data, including OS status and OS time (months), together with the variables used for survival curve construction and Cox regression analyses of prognostic factors. (XLS)

## Abbreviations

AASLD: American Association for the Study of Liver Diseases
AFP: α-fetoprotein
AKI: acute kidney injury
BCLC: Barcelona Clinic Liver Cancer
BMI: body mass index
BSC: best supportive care
CI: confidence interval
COVID-19: coronavirus disease 2019
DG: D-galactose
ERK: extracellular signal-regulated kinase
FIB-4: fibrosis-4 index
HBV: hepatitis B virus
HCC: hepatocellular carcinoma
HBeAg: hepatitis B e antigen
HBsAg: hepatitis B surface antigen
HE: hepatic encephalopathy
HR: hazard ratio
5-HT: 5-hydroxytryptamine
5-HTR: 5-hydroxytryptamine receptor
5-HT3RA: 5-hydroxytryptamine 3 receptor antagonist
IL-1β: interleukin-1β
INVR: Index of Nausea, Vomiting, and Retching
IQR: interquartile range
JNK: c-jun N-terminal kinas
MAPK: mitogen-activated protein kinases
mALBI: Modified Albumin-Bilirubin
α7nAChR: α-7 nicotinic acetylcholine receptor
OS: overall survival
PSM: propensity score matching
SBP: spontaneous bacterial peritonitis
SIRT1: sirtuin 1
TNF-α: tumor necrosis factor
VIF: variance inflation factor
