# Supplementary material for: Association of 5-hydroxytryptamine 3 receptor antagonists with prognosis in patients with advanced hepatocellular carcinoma: A propensity score-matched study
Source: PLoS One. 2026 Oct 8;21(10):e0359798. doi: 10.1371/journal.pone.0359798 (PMC13649010; doi:10.1371/journal.pone.0359798)
Supplement: Supplementary file 1 [file pone.0359798.s001.docx]

**Supplementary data**

**Table S1. Baseline characteristics and laboratory data in patients with and without 5-HT3RAs treatment.**

| Variables | 5-HT3RA User (n = 102) | 5-HT3RA Nonuser (n = 1039) | *p* value |
| --- | --- | --- | --- |
| Age (years), mean ± SD | 55.46 ± 13.42 | 57.28 ± 12.22 | 0.155 |
| Sex |  |  | 0.220 |
| Male, n (%) | 82 (75.2) | 899 (79.7) |  |
| Female, n (%) | 26 (25.5) | 206 (19.8) |  |
| BMI, mean ± SD | 22.53 ± 2.59 | 22.50 ± 3.15 | 0.925 |
| Etiology |  |  | 0.375 |
| HBV, n (%) | 81 (79.4) | 863(83.1) |  |
| HCV, n (%) | 7 (6.9) | 32 (3.1) |  |
| Dual HBV-HCV, n (%) | 3 (2.9) | 39 (3.8) |  |
| ALD, n (%) | 0 (0.0) | 10 (1.0) |  |
| NAFLD, n (%) | 0 (0.0) | 1 (0.1) |  |
| AILD, n (%) | 0 (0.0) | 6 (0.6) |  |
| Others, n (%) | 11 (10.8) | 88 (8.5) |  |
| Hypertension, n (%) | 13 (12.7) | 144 (13.9) | 0.872 |
| Diabetes mellitus, n (%) | 8 (7.8) | 128 (12.3) | 0.241 |
| Smoking, n (%) | 48 (47.1) | 466 (44.9) | 0.746 |
| Alcohol use, n (%) | 16 (15.7) | 180 (17.3) | 0.779 |
| Family history of HCC, n (%) | 6 (5.9) | 55 (5.3) | 0.983 |
| Complication |  |  |  |
| Ascites, n (%) | 35 (34.3) | 328 (31.6) | 0.648 |
| SBP, n (%) | 17 (16.7) | 102 (9.8) | 0.047 |
| HE, n (%) | 3 (2.9) | 34 (3.3) | 1.000 |
| Variceal bleeding, n (%) | 8 (7.8) | 53 (5.1) | 0.345 |
| INVR total score, median (IQR) | 12.00 (9.00-14.00) | 6.00 (4.00-9.00) | <0.001 |
| FIB-4 |  |  | 0.163 |
| F0-F2 | 2 (2.0) | 70 (6.7) |  |
| F3, F4 | 76 (74.5) | 727 (70.0) |  |
| mALBI grade |  |  | 0.003 |
| 1, n (%) | 7 (6.9) | 139 (13.4) |  |
| 2a, n (%) | 9 (8.8) | 199 (19.2) |  |
| 2b, n (%) | 56 (54.9) | 496 (47.7) |  |
| 3, n (%) | 30 (29.4) | 205 (19.7) |  |
| BCLC stage |  |  | 0.719 |
| C, n (%) | 23 (22.5) | 213 (20.5) |  |
| D, n (%) | 79 (77.5) | 826 (79.5) |  |
| AFP (ng/mL), median (IQR) | 265.50 (16.48-20011.50) | 241.40 (8.00-6667.50) | 0.266 |
| 5-HT3RAs |  |  |  |
| Ondansetron, n (%) | 47 (46.1) |  |  |
| Tropisetron, n (%) | 46 (45.1) |  |  |
| Palonosetron, n (%) | 11 (10.8) |  |  |
| Granisetron, n (%) | 10 (9.8) |  |  |

Abbreviations: 5-HT3RA, 5-HT3 receptor antagonist; BMI, body mass index; HBV, hepatitis B virus; HCV, hepatitis C virus; ALD, alcohol-related liver disease; NAFLD, nonalcoholic fatty liver disease; AILD, autoimmune liver disease; SBP, spontaneous bacterial peritonitis; HE, hepatic encephalopathy; INVR, index of nausea, vomiting, and retching; FIB-4, fibrosis-4 index; mALBI, modified albumin–bilirubin grade; BCLC, Barcelona Clinic Liver Cancer; AFP, α-fetoprotein.

**Table S2. Baseline characteristics and laboratory data in patients with and without 5-HT3RAs treatment after propensity score matching.**

| Variables | 5-HT3RA User (n = 89) | 5-HT3RA Nonuser (n = 161) | *p* value |
| --- | --- | --- | --- |
| Age (years), mean ± SD | 55.78 ± 13.80 | 57.39 ± 12.02 | 0.338 |
| Sex |  |  |  |
| Male, n (%) | 67 (75.3) | 118 (73.3) | 0.847 |
| Female, n (%) | 22 (24.7) | 43 (26.7) |  |
| BMI, mean ± SD | 22.43 ± 2.68 | 22.91 ± 2.89 | 0.203 |
| Etiology |  |  | 0.392 |
| HBV, n (%) | 69 (77.5) | 136 (84.5) |  |
| HCV, n (%) | 7 (7.9) | 7 (4.3) |  |
| Dual HBV-HCV, n (%) | 3 (3.4) | 7 (4.3) |  |
| ALD, n (%) | 0 (0.0) | 1 (0.6) |  |
| AILD, n (%) | 0 (0.0) | 0 (0.0) |  |
| Others, n (%) | 10 (11.2) | 10 (6.2) |  |
| Hypertension, n (%) | 15 (16.9) | 23 (14.3) | 0.721 |
| Diabetes mellitus, n (%) | 8 (9.0) | 10 (6.2) | 0.577 |
| Smoking, n (%) | 46 (51.7) | 59 (36.6) | 0.030 |
| Alcohol use, n (%) | 15 (16.9) | 31 (19.3) | 0.765 |
| Family history of HCC, n (%) | 4 (4.5) | 10 (6.2) | 0.781 |
| Complication |  |  |  |
| Ascites, n (%) | 30 (33.7) | 49 (30.4) | 0.696 |
| SBP, n (%) | 13 (14.6) | 24 (14.9) | 1.000 |
| HE, n (%) | 2 (2.2) | 6 (3.7) | 0.794 |
| Variceal bleeding, n (%) | 6 (6.7) | 10 (6.2) | 1.000 |
| INVR total score, median (IQR) | 11.00 (9.00-13.00) | 11.00 (8.00-13.00) | 0.441 |
| FIB-4 |  |  | 0.597 |
| F0-F2 | 2 (2.2) | 7 (4.3) |  |
| F3, F4 | 65 (73.0) | 110 (68.3) |  |
| mALBI grade |  |  | 0.487 |
| 1, n (%) | 7 (7.9) | 19 (11.8) |  |
| 2a, n (%) | 8 (9.0) | 19 (11.8) |  |
| 2b, n (%) | 51 (57.3) | 77 (47.8) |  |
| 3, n (%) | 23 (25.8) | 46 (28.6) |  |
| BCLC stage |  |  | 1.000 |
| C, n (%) | 20 (22.5) | 36 (22.4) |  |
| D, n (%) | 69 (77.5) | 125 (77.6) |  |
| AFP (ng/mL), median (IQR) | 648.00 (11.64-23396.00) | 127.50 (8.00-4997.00) | 0.100 |
| 5-HT3RAs |  |  |  |
| Ondansetron, n (%) | 38 (42.7) |  |  |
| Tropisetron, n (%) | 43 (48.3) |  |  |
| Palonosetron, n (%) | 10 (11.2) |  |  |
| Granisetron, n (%) | 10 (11.2) |  |  |

Abbreviations: 5-HT3RA, 5-HT3 receptor antagonist; BMI, body mass index; HBV, hepatitis B virus; HCV, hepatitis C virus; ALD, alcohol-related liver disease; NAFLD, nonalcoholic fatty liver disease; AILD, autoimmune liver disease; SBP, spontaneous bacterial peritonitis; HE, hepatic encephalopathy; INVR, index of nausea, vomiting, and retching; FIB-4, fibrosis-4 index; mALBI, modified albumin–bilirubin grade; BCLC, Barcelona Clinic Liver Cancer; AFP, alpha-fetoprotein.

**Table S3. Univariate and multivariate Cox regression analyses of variables and overall survival in the patients before propensity score matching.**

|  | Univariate | | | Multivariate | | |
| --- | --- | --- | --- | --- | --- | --- |
|  | HR | 95% CI | *p* value | HR | 95% CI | *p* value |
| Sex |  |  |  |  |  |  |
| Female | Reference |  |  | Reference |  |  |
| Male | 1.167 | 0.995-1.369 | 0.058 | 1.169 | 0.994-1.374 | 0.059 |
| Hypertension | 0.947 | 0.787-1.139 | 0.561 |  |  |  |
| Diabetes mellitus | 0.916 | 0.751-1.117 | 0.387 |  |  |  |
| Smoking | 1.015 | 0.894-1.154 | 0.814 |  |  |  |
| Alcohol use | 0.996 | 0.842-1.178 | 0.963 |  |  |  |
| Family history of HCC | 1.123 | 0.853-1.478 | 0.407 |  |  |  |
| Complication |  |  |  |  |  |  |
| Ascites | 1.240 | 1.083-1.420 | 0.002 | 1.022 | 0.878-1.190 | 0.781 |
| SBP | 1.319 | 1.077-1.616 | 0.007 | 1.174 | 0.938-1.468 | 0.161 |
| HE | 1.266 | 0.903-1.774 | 0.172 | 0.948 | 0.661-1.358 | 0.770 |
| Variceal bleeding | 1.258 | 0.956-1.656 | 0.101 | 1.143 | 0.863-1.516 | 0.351 |
| FIB-4 |  |  |  |  |  |  |
| F0-F2 | Reference |  |  | Reference |  |  |
| F3, F4 | 1.445 | 1.087-1.920 | 0.011 | 1.336 | 0.999-1.786 | 0.051 |
| mALBI grade |  |  |  |  |  |  |
| 1 | Reference |  |  | Reference |  |  |
| 2a | 1.207 | 0.949-1.536 | 0.125 | 1.145 | 0.898-1.460 | 0.276 |
| 2b | 1.482 | 1.203-1.826 | <0.001 | 1.261 | 1.014-1.568 | 0.037 |
| 3 | 1.569 | 1.241-1.982 | <0.001 | 1.330 | 1.030-1.716 | 0.029 |
| BCLC stage |  |  |  |  |  |  |
| C | Reference |  |  | Reference |  |  |
| D | 1.690 | 1.430-1.996 | <0.001 | 1.579 | 1.333-1.871 | <0.001 |
| AFP (ng/mL) |  |  |  |  |  |  |
| <400 | Reference |  |  | Reference |  |  |
| ≥400 | 1.296 | 1.141-1.473 | <0.001 | 1.207 | 1.059-1.376 | 0.005 |
| 5-HT3RAs |  |  |  |  |  |  |
| Nonuser | Reference |  |  | Reference |  |  |
| User | 0.742 | 0.589-0.935 | 0.011 | 0.690 | 0.546-0.871 | 0.002 |

Abbreviations: HR, hazard ratio; CI, confidence interval; SBP, spontaneous bacterial peritonitis; HE, hepatic encephalopathy; FIB-4, fibrosis-4 index; mALBI, modified albumin–bilirubin grade; BCLC, Barcelona Clinic Liver Cancer; AFP, α-fetoprotein; 5-HT3RA, 5-HT3 receptor antagonist.

**Table S4. Baseline characteristics in HBV-related HCC patients with and without 5-HT3RAs treatment.**

| Variables | 5-HT3RA User (n = 81) | 5-HT3RA Nonuser (n = 863) | *p* value |
| --- | --- | --- | --- |
| Age (years), mean ± SD | 53.32 ± 12.61 | 55.79 ± 11.85 | 0.075 |
| Sex |  |  | 0.017 |
| Male, n (%) | 60 (74.1) | 733 (84.9) |  |
| Female, n (%) | 21 (25.9) | 130 (15.1) |  |
| BMI, mean ± SD | 22.54 ± 2.61 | 22.46 ± 3.10 | 0.830 |
| Hypertension, n (%) | 12 (14.8) | 93 (10.8) | 0.357 |
| Diabetes mellitus, n (%) | 6 (7.4) | 81 (9.4) | 0.698 |
| Smoking, n (%) | 36 (44.4) | 403 (46.7) | 0.785 |
| Alcohol use, n (%) | 12 (14.8) | 159 (18.4) | 0.512 |
| Family history of HCC, n (%) | 5 (6.2) | 52 (6.0) | 1.000 |
| Complication |  |  |  |
| Ascites, n (%) | 32 (39.5) | 264 (30.6) | 0.126 |
| SBP, n (%) | 15 (18.5) | 88 (10.2) | 0.035 |
| HE, n (%) | 7 (8.6) | 44 (5.1) | 0.275 |
| Variceal bleeding, n (%) | 1 (1.2) | 25 (2.9) | 0.604 |
| INVR total score, median (IQR) | 11.00 (9.00-14.00) | 6.00 (4.00-9.00) | <0.001 |
| FIB-4 |  |  | 0.151 |
| F0-F2 | 1 (1.2) | 57 (6.6) |  |
| F3, F4 | 61 (75.3) | 603 (69.9) |  |
| mALBI |  |  | 0.004 |
| 1, n (%) | 5 (6.2) | 121 (14.0) |  |
| 2a, n (%) | 6 (7.4) | 162 (18.8) |  |
| 2b, n (%) | 47 (58.0) | 416 (48.2) |  |
| 3, n (%) | 23 (28.4) | 164 (19.0) |  |
| BCLC stage |  |  | 0.637 |
| C, n (%) | 15 (18.5) | 185 (21.4) |  |
| D, n (%) | 66(81.5) | 678(78.6) |  |
| AFP (ng/mL), median (IQR) | 648.00 (44.80-24797.00) | 337.30 (9.50-8368.00) | 0.128 |
| HBsAg (IU/mL), median (IQR) | 161.26 (17.50-1484.62) | 128.98 (16.88-1484.62) | 0.396 |
| HBeAg positive, n (%) | 16 (19.8) | 156 (18.1) | 0.823 |
| Antiviral agent, n (%) | 47 (58.0) | 407 (47.2) | 0.079 |
| ETV, n (%) | 39 (48.1) | 349 (40.4) | 0.219 |
| TAF, n (%) | 2 (2.5) | 6 (0.7) | 0.302 |
| TDF, n (%) | 3 (3.7) | 18 (2.1) | 0.582 |
| ADV, n (%) | 6 (7.4) | 19 (2.2) | 0.015 |
| LAM, n (%) | 3 (3.7) | 34 (3.9) | 1.000 |
| LdT, n (%) | 0 (0.0) | 4 (0.5) | 1.000 |
| IFN, n (%) | 0 (0.0) | 1 (0.1) | 1.000 |
| 5-HT3RAs |  |  |  |
| Ondansetron, n (%) | 36 (44.4) |  |  |
| Tropisetron, n (%) | 35 (43.2) |  |  |
| Palonosetron, n (%) | 11 (13.6) |  |  |
| Granisetron, n (%) | 9 (11.1) |  |  |

Abbreviations: HBV, hepatitis B virus; HCC, hepatocellular carcinoma; 5-HT3RA, 5-HT3 receptor antagonist; BMI, body mass index; SBP, spontaneous bacterial peritonitis; HE, hepatic encephalopathy; INVR, index of nausea, vomiting, and retching; FIB-4, fibrosis-4 index; mALBI, modified albumin–bilirubin grade; BCLC, Barcelona Clinic Liver Cancer; AFP, alpha-fetoprotein; ETV, entecavir; TAF, tenofovir alafenamide; TDF, tenofovir disoproxil fumarate; ADV, adefovir; LAM, lamivudine; LdT, telbivudine; IFN, interferon.

**Table S5. Baseline characteristics in HBV-related HCC patients with and without 5-HT3RA treatment after propensity score matching.**

| Variables | 5-HT3RA User (n = 80) | 5-HT3RA Nonuser (n = 158) | *p* value |
| --- | --- | --- | --- |
| Age (years), mean ± SD | 53.28 ± 12.69 | 55.99 ± 11.07 | 0.091 |
| Sex |  |  | 1.000 |
| Male, n (%) | 60 (75.0) | 119 (75.3) |  |
| Female, n (%) | 20 (25.0) | 39 (24.7) |  |
| BMI, mean ± SD | 22.52 ± 2.62 | 22.77 ± 3.29 | 0.557 |
| Hypertension, n (%) | 12 (15.0) | 19 (12.0) | 0.660 |
| Diabetes mellitus, n (%) | 5 (6.2) | 19 (12.0) | 0.242 |
| Smoking, n (%) | 36 (45.0) | 59 (37.3) | 0.318 |
| Alcohol use, n (%) | 12 (15.0) | 30 (19.0) | 0.560 |
| Family history of HCC, n (%) | 5 (6.2) | 9 (5.7) | 1.000 |
| Complication |  |  |  |
| Ascites, n (%) | 31 (38.8) | 59 (37.3) | 0.944 |
| SBP, n (%) | 15 (18.8) | 24 (15.2) | 0.606 |
| HE, n (%) | 6 (7.5) | 14 (8.9) | 0.912 |
| Variceal bleeding, n (%) | 1 (1.2) | 1 (0.6) | 1.000 |
| INVR total score, median (IQR) | 11.00 (9.00-14.00) | 11.00 (8.25-13.00) | 0.089 |
| FIB-4 |  |  | 0.883 |
| F0-F2 | 1 (1.2) | 1 (0.6) |  |
| F3, F4 | 60 (75.0) | 120 (75.9) |  |
| mALBI |  |  | 0.975 |
| 1, n (%) | 5 (6.2) | 10 (6.3) |  |
| 2a, n (%) | 6 (7.5) | 10 (6.3) |  |
| 2b, n (%) | 46 (57.5) | 95 (60.1) |  |
| 3, n (%) | 23 (28.7) | 43 (27.2) |  |
| BCLC stage |  |  | 0.742 |
| C, n (%) | 15 (18.8) | 34 (21.5) |  |
| D, n (%) | 65 (81.2) | 124 (78.5) |  |
| AFP (ng/mL), median (IQR) | 586.50 (42.09-25307.00) | 311.15 (8.89-8635.00) | 0.261 |
| HBsAg (IU/mL), median (IQR) | 153.04 (15.28-1484.62) | 130.79 (81.14-1484.62) | 0.826 |
| HBeAg positive, n (%) | 15 (18.8) | 30 (19.0) | 1.000 |
| Antiviral agent, n (%) | 46 (57.5) | 81 (51.3) | 0.439 |
| ETV, n (%) | 39 (48.8) | 72 (45.6) | 0.744 |
| TAF, n (%) | 2 (2.5) | 0 (0.0) | 0.213 |
| TDF, n (%) | 3 (3.8) | 4 (2.5) | 0.905 |
| ADV, n (%) | 6 (7.5) | 3 (1.9) | 0.075 |
| LAM, n (%) | 2 (2.5) | 7 (4.4) | 0.706 |
| LdT, n (%) | 0 (0.0) | 0 (0.0) |  |
| IFN, n (%) | 0 (0.0) | 0 (0.0) |  |
| 5-HT3RAs |  |  |  |
| Ondansetron, n (%) | 35 (43.8) |  |  |
| Tropisetron, n (%) | 35 (43.8) |  |  |
| Palonosetron, n (%) | 11 (13.8) |  |  |
| Granisetron, n (%) | 9 (11.2) |  |  |

Abbreviations: HBV, hepatitis B virus; HCC, hepatocellular carcinoma; 5-HT3RA, 5-HT3 receptor antagonist; BMI, body mass index; SBP, spontaneous bacterial peritonitis; HE, hepatic encephalopathy; INVR, index of nausea, vomiting, and retching; FIB-4, fibrosis-4 index; mALBI, modified albumin–bilirubin grade; BCLC, Barcelona Clinic Liver Cancer; AFP, alpha-fetoprotein; ETV, entecavir; TAF, tenofovir alafenamide; TDF, tenofovir disoproxil fumarate; ADV, adefovir; LAM, lamivudine; LdT, telbivudine; IFN, interferon

**Table S6. Univariate and multivariate Cox regression analyses of variables and overall survival in HBV-related hepatocellular carcinoma patients before propensity score matching.**

|  | Univariate |  |  | Multivariate |  |  |
| --- | --- | --- | --- | --- | --- | --- |
|  | HR | 95% CI | *p* value | HR | 95% CI | *p* value |
| Sex |  |  |  |  |  |  |
| Female | Reference |  |  | Reference |  |  |
| Male | 1.212 | 0.997-1.474 | 0.053 | 1.203 | 0.987-1.466 | 0.067 |
| Complication |  |  |  |  |  |  |
| Ascites | 1.241 | 1.068-1.440 | 0.005 | 1.077 | 0.911-1.272 | 0.385 |
| SBP | 1.331 | 1.070-1.657 | 0.010 | 1.215 | 0.958-1.540 | 0.108 |
| HE | 1.174 | 0.782-1.762 | 0.440 |  |  |  |
| Variceal bleeding | 1.164 | 0.862-1.573 | 0.322 |  |  |  |
| FIB-4 |  |  |  |  |  |  |
| F0-F2 | Reference |  |  | Reference |  |  |
| F3, F4 | 1.504 | 1.095-2.065 | 0.011 | 1.361 | 0.986-1.879 | 0.061 |
| mALBI grade |  |  |  |  |  |  |
| 1 | Reference |  |  | Reference |  |  |
| 2a | 1.151 | 0.885-1.495 | 0.294 | 1.134 | 0.871-1.477 | 0.350 |
| 2b | 1.473 | 1.177-1.843 | 0.001 | 1.312 | 1.040-1.656 | 0.022 |
| 3 | 1.590 | 1.234-2.051 | <0.001 | 1.427 | 1.081-1.883 | 0.012 |
| BCLC stage |  |  |  |  |  |  |
| C | Reference |  |  | Reference |  |  |
| D | 1.693 | 1.413-2.028 | <0.001 | 1.599 | 1.331-1.920 | <0.001 |
| AFP (ng/mL) |  |  |  |  |  |  |
| <400 | Reference |  |  | Reference |  |  |
| ≥400 | 1.285 | 1.118-1.478 | <0.001 | 1.219 | 1.056-1.407 | 0.007 |
| HBeAg positive | 0.954 | 0.795-1.150 | 0.616 |  |  |  |
| Antiviral agent | 0.880 | 0.765-1.012 | 0.073 | 0.809 | 0.701-0.935 | 0.004 |
| ETV | 0.890 | 0.772-1.027 | 0.110 | 0.803 | 0.693-0.930 | 0.004 |
| TAF | 0.640 | 0.266-1.542 | 0.320 | 0.501 | 0.206-1.220 | 0.128 |
| TDF | 0.589 | 0.333-1.042 | 0.069 | 0.584 | 0.328-1.041 | 0.068 |
| ADV | 0.863 | 0.565-1.319 | 0.496 | 0.931 | 0.587-1.476 | 0.761 |
| LAM | 1.034 | 0.729-1.465 | 0.852 | 0.970 | 0.665-1.415 | 0.876 |
| LdT | 1.560 | 0.584-4.169 | 0.375 | 1.638 | 0.609-4.402 | 0.328 |
| IFN | 4.044 | 0.568-28.806 | 0.163 | 3.073 | 0.427-22.094 | 0.265 |
| 5-HT3RA |  |  |  |  |  |  |
| Nonuser | Reference |  |  | Reference |  |  |
| User | 0.740 | 0.590-0.930 | 0.011 | 0.611 | 0.469-0.797 | <0.001 |

Abbreviations: HBV, hepatitis B virus; HCC, hepatocellular carcinoma; HR, hazard ratio; CI, confidence interval; SBP, spontaneous bacterial peritonitis; HE, hepatic encephalopathy; FIB-4, fibrosis-4 index; mALBI, modified albumin–bilirubin grade; BCLC, Barcelona Clinic Liver Cancer; AFP, α-fetoprotein; ETV, entecavir; TAF, tenofovir alafenamide; TDF, tenofovir disoproxil fumarate; ADV, adefovir; LAM, lamivudine; LdT, telbivudine; IFN, interferon; 5-HT3RA, 5-HT3 receptor antagonist.

**Table S7. Baseline characteristics and laboratory data in patients with and without the use of ondansetron.**

| Variables | Ondansetron User (n = 47) | Ondansetron Nonuser (n = 1039) | *p* value |
| --- | --- | --- | --- |
| Age (years), mean ± SD | 55.78 ± 13.54 | 57.28 ± 12.22 | 0.415 |
| Sex |  |  | 0.261 |
| Male, n (%) | 34 (72.3) | 833 (80.2) |  |
| Female, n (%) | 13 (27.7) | 206 (19.8) |  |
| BMI, mean ± SD | 22.67 ± 2.83 | 22.5 ± 3.15 | 0.832 |
| Etiology |  |  | 0.735 |
| HBV, n (%) | 36 (76.6) | 863 (83.1) |  |
| HCV, n (%) | 3 (6.4) | 32 (3.1) |  |
| Dual HBV-HCV, n (%) | 3 (6.4) | 39 (3.8) |  |
| ALD, n (%) | 0 (0.0) | 10 (1.0) |  |
| NAFLD, n (%) | 0 (0.0) | 1 (0.1) |  |
| AILD, n (%) | 0 (0.0) | 6 (0.6) |  |
| Others, n (%) | 5(10.6) | 88 (8.5) |  |
| Hypertension, n (%) | 8 (17.0) | 141 (13.6) | 0.649 |
| Diabetes mellitus, n (%) | 3 (6.4) | 127 (12.2) | 0.329 |
| Smoking, n (%) | 22 (46.8) | 466 (44.9) | 0.909 |
| Alcohol use, n (%) | 7 (14.9) | 180 (17.3) | 0.815 |
| Family history of HCC, n (%) | 5 (10.6) | 55 (5.3) | 0.214 |
| Complication |  |  |  |
| Ascites, n (%) | 21 (44.2) | 328 (31.6) | 0.214 |
| SBP, n (%) | 13 (27.7) | 102 (9.8) | <0.001 |
| HE, n (%) | 1 (2.1) | 34 (3.3) | 0.990 |
| Variceal bleeding, n (%) | 5 (10.6) | 53 (5.1) | 0.187 |
| INVR total score, median (IQR) | 12.00 (9.00-15.50) | 6.00 (4.00-9.00) | <0.001 |
| FIB-4 |  |  | 0.141 |
| F0-F2 | 1 (2.1) | 70 (6.7) |  |
| F3, F4 | 39 (83.0) | 727 (70.0) |  |
| mALBI grade |  |  | 0.009 |
| 1, n (%) | 2 (4.3) | 139 (13.4) |  |
| 2a, n (%) | 4 (8.5) | 199 (19.2) |  |
| 2b, n (%) | 24 (51.1) | 496 (47.7) |  |
| 3, n (%) | 17 (36.2) | 205 (19.7) |  |
| BCLC stage |  |  | 0.318 |
| C, n (%) | 13 (27.7) | 213 (20.5) |  |
| D, n (%) | 34 (72.3) | 826 (79.5) |  |
| AFP (ng/mL), median (IQR) | 144.00 (45.55-18655.00) | 241.40 (8.00-6667.50) | 0.403 |

Abbreviations: BMI, body mass index; HBV, hepatitis B virus; HCV, hepatitis C virus; ALD, alcohol-related liver disease; NAFLD, nonalcoholic fatty liver disease; AILD, autoimmune liver disease; SBP, spontaneous bacterial peritonitis; HE, hepatic encephalopathy; INVR, index of nausea, vomiting, and retching; FIB-4, fibrosis-4 index; mALBI, modified albumin–bilirubin grade; BCLC, Barcelona Clinic Liver Cancer; AFP, alpha-fetoprotein.

**Table S8. Baseline characteristics and laboratory data in patients with and without the use of ondansetron after propensity score matching.**

| Variables | Ondansetron User (n = 37) | Ondansetron Nonuser (n = 71) | *p* value |
| --- | --- | --- | --- |
| Age (years), mean ± SD | 57.8 ± 14.44 | 57.15 ± 11.92 | 0.805 |
| Sex |  |  | 0.832 |
| Male, n (%) | 26 (70.3) | 47 (66.2) |  |
| Female, n (%) | 11 (29.7) | 24 (23.8) |  |
| BMI, mean ± SD | 22.54 ± 2.83 | 23.37 ± 3.80 | 0.248 |
| Etiology |  |  | 0.735 |
| HBV, n (%) | 27 (73.0) | 56 (78.9) |  |
| HCV, n (%) | 3 (8.1) | 3 (4.2) |  |
| Dual HBV-HCV, n (%) | 2 (5.4) | 5 (7.0) |  |
| AILD, n (%) | 0 (0.0) | 1 (1.4) |  |
| Others, n (%) | 5 (13.5) | 5 (7.0) |  |
| Hypertension, n (%) | 6 (16.2) | 13 (18.3) | 0.996 |
| Diabetes mellitus, n (%) | 3 (8.1) | 5 (7.0) | 1.000 |
| Smoking, n (%) | 17 (45.9) | 24 (33.8) | 0.305 |
| Alcohol use, n (%) | 3 (8.1) | 11 (15.5) | 0.434 |
| Family history of HCC, n (%) | 3 (8.1) | 8 (11.3) | 0.857 |
| Complication |  |  |  |
| Ascites, n (%) | 14 (37.8) | 29 (40.8) | 0.924 |
| SBP, n (%) | 8 (21.6) | 14 (19.7) | 1.000 |
| HE, n (%) | 4 (10.8) | 9 (12.7) | 1.000 |
| Variceal bleeding, n (%) | 1 (2.7) | 0 (0.0) | 0.739 |
| INVR total score, median (IQR) | 11.00 (8.00-13.00) | 9.00 (7.00-13.00) | 0.528 |
| FIB-4 |  |  | 0.994 |
| F0-F2 | 1 (2.7) | 2 (2.8) |  |
| F3, F4 | 29 (78.4) | 55 (77.5) |  |
| mALBI grade |  |  | 0.820 |
| 1, n (%) | 2 (5.4) | 7 (9.9) |  |
| 2a, n (%) | 3 (8.1) | 6 (8.5) |  |
| 2b, n (%) | 20 (54.1) | 33 (46.5) |  |
| 3, n (%) | 12 (32.4) | 25 (35.2) |  |
| BCLC stage |  |  | 0.580 |
| C, n (%) | 12 (32.4) | 18 (25.4) |  |
| D, n (%) | 25 (67.6) | 53 (74.6) |  |
| AFP (ng/mL), median (IQR) | 324.00 (15.35-20020.00) | 124.00 (8.00-5540.00) | 0.356 |

Abbreviations: BMI, body mass index; HBV, hepatitis B virus; HCV, hepatitis C virus; AILD, autoimmune liver disease; SBP, spontaneous bacterial peritonitis; HE, hepatic encephalopathy; INVR, index of nausea, vomiting, and retching; FIB-4, fibrosis-4 index; mALBI, modified albumin–bilirubin grade; BCLC, Barcelona Clinic Liver Cancer; AFP, alpha-fetoprotein.

**Table S9. Univariate and multivariate Cox regression analyses of variables and overall survival in patients with and without the use of ondansetron before propensity score matching.**

|  | Univariate |  |  | Multivariate |  |  |
| --- | --- | --- | --- | --- | --- | --- |
|  | HR | 95% CI | *p* value | HR | 95% CI | *p* value |
| Sex |  |  |  |  |  |  |
| Female | Reference |  |  | Reference |  |  |
| Male | 1.169 | 0.992-1.378 | 0.062 | 1.158 | 0.974-1.376 | 0.097 |
| Hypertension | 0.940 | 0.778-1.135 | 0.518 |  |  |  |
| Diabetes mellitus | 0.919 | 0.750-1.125 | 0.413 |  |  |  |
| Smoking | 1.007 | 0.883-1.147 | 0.920 |  |  |  |
| Alcohol use | 0.988 | 0.832-1.173 | 0.889 |  |  |  |
| Family history of HCC | 1.087 | 0.823-1.434 | 0.558 |  |  |  |
| Complication |  |  |  |  |  |  |
| Ascites | 1.258 | 1.096-1.445 | 0.001 | 1.055 | 0.901-1.234 | 0.507 |
| SBP | 1.335 | 1.087-1.639 | 0.006 | 1.196 | 0.949-1.509 | 0.129 |
| HE | 1.192 | 0.842-1.687 | 0.323 | 0.942 | 0.648-1.371 | 0.756 |
| Variceal bleeding | 1.202 | 0.906-1.594 | 0.203 | 1.077 | 0.805-1.442 | 0.617 |
| FIB-4 |  |  |  |  |  |  |
| F0-F2 | Reference |  |  | Reference |  |  |
| F3, F4 | 1.472 | 1.104-1.962 | 0.009 | 1.346 | 1.003-1.807 | 0.048 |
| mALBI grade |  |  |  |  |  |  |
| 1 | Reference |  |  | Reference |  |  |
| 2a | 1.202 | 0.942-1.534 | 0.138 | 1.156 | 0.902-1.480 | 0.252 |
| 2b | 1.491 | 1.206-1.843 | <0.001 | 1.264 | 1.011-1.580 | 0.040 |
| 3 | 1.536 | 1.210-1.950 | <0.001 | 1.324 | 1.015-1.727 | 0.038 |
| BCLC stage |  |  |  |  |  |  |
| C | Reference |  |  | Reference |  |  |
| D | 1.695 | 1.43-2.009 | <0.001 | 1.575 | 1.323-1.875 | <0.001 |
| AFP (ng/mL) |  |  |  |  |  |  |
| <400 | Reference |  |  | Reference |  |  |
| ≥400 | 1.336 | 1.173-1.523 | <0.001 | 1.231 | 1.075-1.410 | 0.003 |
| Ondansetron |  |  |  |  |  |  |
| Nonuser | Reference |  |  | Reference |  |  |
| User | 0.793 | 0.571-1.103 | 0.168 | 0.712 | 0.507-1.000 | 0.050 |

Abbreviations: HR, hazard ratio; CI, confidence interval; SBP, spontaneous bacterial peritonitis; HE, hepatic encephalopathy; FIB-4, fibrosis-4 index; mALBI, modified albumin–bilirubin grade; BCLC, Barcelona Clinic Liver Cancer; AFP, α-fetoprotein.

**Table S10. Baseline characteristics and laboratory data in patients with and without the use of tropisetron.**

| Variables | Tropisetron User (n = 46) | Tropisetron Nonuser (n = 1039) | *p* value |
| --- | --- | --- | --- |
| Age (years), mean ± SD | 55.84 ± 13.00 | 57.28 ± 12.22 | 0.437 |
| Sex |  |  | 0.396 |
| Male, n (%) | 34 (73.9) | 833 (80.2) |  |
| Female, n (%) | 12 (26.1) | 206 (19.8) |  |
| BMI, mean ± SD | 21.96 ± 1.91 | 22.5 ± 3.15 | 0.256 |
| Etiology |  |  | 0.162 |
| HBV, n (%) | 35 (76.1) | 863 (83.1) |  |
| HCV, n (%) | 4 (8.7) | 32 (3.1) |  |
| Dual HBV-HCV, n (%) | 0 (0.0) | 39 (3.8) |  |
| ALD, n (%) | 0 (0.0) | 10 (1.0) |  |
| NAFLD, n (%) | 0 (0.0) | 1 (0.1) |  |
| AILD, n (%) | 0 (0.0) | 6 (0.6) |  |
| Others, n (%) | 7 (15.2) | 88 (8.5) |  |
| Hypertension, n (%) | 4 (8.7) | 141 (13.6) | 0.466 |
| Diabetes mellitus, n (%) | 4 (8.7) | 127 (12.2) | 0.626 |
| Smoking, n (%) | 20 (43.5) | 466 (44.9) | 0.975 |
| Alcohol use, n (%) | 7 (15.2) | 180 (17.3) | 0.864 |
| Family history of HCC, n (%) | 0 (0.0) | 55 (5.3) | 0.208 |
| Complication |  |  |  |
| Ascites, n (%) | 10 (21.7) | 328 (31.6) | 0.213 |
| SBP, n (%) | 1 (2.2) | 102 (9.8) | 0.141 |
| HE, n (%) | 1 (2.2) | 34 (3.3) | 1.000 |
| Variceal bleeding, n (%) | 2 (4.3) | 53 (5.1) | 1.000 |
| INVR total score, median (IQR) | 11.00 (9.00-12.75) | 6.00 (4.00-9.00) | <0.001 |
| FIB-4 |  |  | 0.478 |
| F0-F2 | 2 (4.3) | 70 (6.7) |  |
| F3, F4 | 30 (65.2) | 727 (70.0) |  |
| mALBI grade |  |  | 0.766 |
| 1, n (%) | 6 (13.0) | 139 (13.4) |  |
| 2a, n (%) | 6 (13.0) | 199 (19.2) |  |
| 2b, n (%) | 24 (52.2) | 496 (47.7) |  |
| 3, n (%) | 10 (21.7) | 205 (19.7) |  |
| BCLC stage |  |  | 0.709 |
| C, n (%) | 11 (23.9) | 213 (20.5) |  |
| D, n (%) | 35 (76.1) | 826 (79.5) |  |
| AFP (ng/mL), median (IQR) | 142.55 (6.68-15926.00) | 241.40 (8.00-6667.50) | 0.771 |

Abbreviations: BMI, body mass index; HBV, hepatitis B virus; HCV, hepatitis C virus; ALD, alcohol-related liver disease; NAFLD, nonalcoholic fatty liver disease; AILD, autoimmune liver disease; SBP, spontaneous bacterial peritonitis; HE, hepatic encephalopathy; INVR, index of nausea, vomiting, and retching; FIB-4, fibrosis-4 index; mALBI, modified albumin–bilirubin grade; BCLC, Barcelona Clinic Liver Cancer; AFP, alpha-fetoprotein.

**Table S11. Baseline characteristics and laboratory data in patients with and without the use of tropisetron after propensity score matching.**

| Variables | Tropisetron User (n = 38) | Tropisetron Nonuser (n = 76) | *p* value |
| --- | --- | --- | --- |
| Age (years), mean ± SD | 56.69 ± 13.75 | 57.47 ± 12.61 | 0.763 |
| Sex |  |  | 0.941 |
| Male, n (%) | 27 (71.1) | 56 (73.7) |  |
| Female, n (%) | 11 (28.9) | 20 (26.3) |  |
| BMI, mean ± SD | 21.99 ± 1.91 | 22.53 ± 3.62 | 0.371 |
| Etiology |  |  | 0.471 |
| HBV, n (%) | 32 (84.2) | 62 (81.6) |  |
| HCV, n (%) | 2 (5.3) | 1 (1.3) |  |
| Dual HBV-HCV, n (%) | 0 (0.0) | 1 (1.3) |  |
| Others, n (%) | 4 (10.5) | 12 (15.8) |  |
| Hypertension, n (%) | 4 (10.5) | 7 (9.2) | 1.000 |
| Diabetes mellitus, n (%) | 4 (10.5) | 4 (5.3) | 0.517 |
| Smoking, n (%) | 15 (39.5) | 28 (36.8) | 0.946 |
| Alcohol use, n (%) | 5 (13.2) | 15 (19.7) | 0.542 |
| Family history of HCC, n (%) | 0 (0.0) | 2 (2.6) | 0.801 |
| Complication |  |  |  |
| Ascites, n (%) | 10 (26.3) | 20 (26.3) | 1.000 |
| SBP, n (%) | 1 (2.6) | 4 (5.3) | 0.872 |
| HE, n (%) | 0 (0.0) | 3 (3.9) | 0.535 |
| Variceal bleeding, n (%) | 1 (2.6) | 0 (0.0) | 0.723 |
| INVR total score, median (IQR) | 10.50 (8.25-12.00) | 10.00 (8.00-12.00) | 0.993 |
| FIB-4 |  |  | 0.962 |
| F0-F2 | 2 (5.3) | 5 (6.6) |  |
| F3, F4 | 25 (65.8) | 49 (64.5) |  |
| mALBI grade |  |  | 0.749 |
| 1, n (%) | 5 (13.2) | 9 (11.8) |  |
| 2a, n (%) | 6 (15.8) | 8 (10.5) |  |
| 2b, n (%) | 19 (50.0) | 37 (48.7) |  |
| 3, n (%) | 8 (21.1) | 22 (28.9) |  |
| BCLC stage |  |  | 0.338 |
| C, n (%) | 9 (23.7) | 11 (14.5) |  |
| D, n (%) | 29 (76.3) | 65 (85.5) |  |
| AFP (ng/mL), median (IQR) | 154.15 (6.68-18020.00) | 99.91 (8.00-6887.00) | 0.682 |

Abbreviations: BMI, body mass index; HBV, hepatitis B virus; HCV, hepatitis C virus; SBP, spontaneous bacterial peritonitis; HE, hepatic encephalopathy; INVR, index of nausea, vomiting, and retching; FIB-4, fibrosis-4 index; mALBI, modified albumin–bilirubin grade; BCLC, Barcelona Clinic Liver Cancer; AFP, alpha-fetoprotein.

**Table S12. Univariate and multivariate Cox regression analyses of variables and overall survival in patients with and without the use of tropisetron before propensity score matching.**

|  | Univariate | | | Multivariate | | |
| --- | --- | --- | --- | --- | --- | --- |
|  | HR | 95% CI | *p* value | HR | 95% CI | *p* value |
| Sex |  |  |  |  |  |  |
| Female | Reference |  |  | Reference |  |  |
| Male | 1.167 | 0.991-1.375 | 0.065 | 1.188 | 1.006-1.403 | 0.042 |
| Hypertension | 0.934 | 0.771-1.131 | 0.481 |  |  |  |
| Diabetes mellitus | 0.894 | 0.730-1.095 | 0.278 |  |  |  |
| Smoking | 1.003 | 0.880-1.143 | 0.966 |  |  |  |
| Alcohol use | 0.993 | 0.836-1.179 | 0.935 |  |  |  |
| Family history of HCC | 1.158 | 0.871-1.541 | 0.312 |  |  |  |
| Complication |  |  |  |  |  |  |
| Ascites | 1.262 | 1.098-1.451 | 0.001 | 1.025 | 0.876-1.200 | 0.754 |
| SBP | 1.401 | 1.129-1.738 | 0.002 | 1.220 | 0.960-1.551 | 0.104 |
| HE | 1.225 | 0.865-1.734 | 0.253 | 0.889 | 0.612-1.290 | 0.535 |
| Variceal bleeding | 1.212 | 0.906-1.621 | 0.195 | 1.097 | 0.814-1.477 | 0.544 |
| FIB-4 |  |  |  |  |  |  |
| F0-F2 | Reference |  |  | Reference |  |  |
| F3, F4 | 1.471 | 1.106-1.957 | 0.008 | 1.354 | 1.012-1.811 | 0.041 |
| mALBI grade |  |  |  |  |  |  |
| 1 | Reference |  |  | Reference |  |  |
| 2a | 1.210 | 0.951-1.540 | 0.121 | 1.148 | 0.900-1.466 | 0.266 |
| 2b | 1.463 | 1.186-1.805 | <0.001 | 1.227 | 0.985-1.528 | 0.068 |
| 3 | 1.637 | 1.291-2.075 | <0.001 | 1.343 | 1.037-1.739 | 0.026 |
| BCLC stage |  |  |  |  |  |  |
| C | Reference |  |  | Reference |  |  |
| D | 1.687 | 1.423-2.001 | <0.001 | 1.589 | 1.336-1.889 | <0.001 |
| AFP (ng/mL) |  |  |  |  |  |  |
| <400 | Reference |  |  | Reference |  |  |
| ≥400 | 1.280 | 1.123-1.458 | <0.001 | 1.185 | 1.037-1.355 | 0.013 |
| Tropisetron |  |  |  |  |  |  |
| Nonuser | Reference |  |  | Reference |  |  |
| User | 0.720 | 0.518-1.001 | 0.051 | 0.718 | 0.516-1.000 | 0.050 |

Abbreviations: HR, hazard ratio; CI, confidence interval; SBP, spontaneous bacterial peritonitis; HE, hepatic encephalopathy; FIB-4, fibrosis-4 index; mALBI, modified albumin–bilirubin grade; BCLC, Barcelona Clinic Liver Cancer; AFP, α-fetoprotein.
